# Single-cell profiling uncovers synovial fibroblast subpopulations associated with chondrocyte injury in osteoarthritis

**DOI:** 10.3389/fendo.2024.1479909

**Published:** 2024-12-10

**Authors:** Zezhong Liu, Yongqi Sun, Jiaoyi Pan, Kechun Guo, Zhi Tang, Xiaofeng Wang

**Affiliations:** ^1^ Spinal Surgery, Zhejiang Chinese Medical University Affiliated Wenzhou Hospital of Integrated Traditional Chinese and Western Medicine, Wenzhou, Zhejiang, China; ^2^ The Second Clinical Medical College, Zhejiang Chinese Medical University, Hangzhou, Zhejiang, China; ^3^ Bonesetting Center, Xiangtan Chinese Medicine Hospital, Xiangtan, Hunan, China

**Keywords:** osteoarthritis, synovial fibroblasts, chondrocytes, interaction, pathogenesis

## Abstract

**Background:**

Chondrocytes and synovial cells participate in the pathogenesis of osteoarthritis (OA). Nonetheless, the interactions and correlations between OA synovial cells and chondrocytes remain unclear. This study aims to elucidate the interactions and correlations between OA synovial cells and chondrocytes, so as to deepen understanding of OA pathogenesis.

**Methods:**

Single-cell sequencing analysis was employed to analyze clusters of synovial and chondrocyte cells within the OA dataset. Through cell interaction analysis, the potential interactions between these two cell types were further explored. Differential gene expression analysis was used to examine the differences among synovial-related cell clusters.

**Results:**

The study identified specific characteristics of synovial fibroblasts through single-cell sequencing analysis. Subsequent cell interaction analysis revealed interactions and correlations between synovial fibroblast clusters and cell clusters in both damaged and non-damaged cartilages. *CILP*+ fibroblasts showed significant interactions with non-damaged chondrocytes, while *POSTN*+ fibroblasts exhibited significant interactions with damaged chondrocytes. Furthermore, differential gene expression analysis revealed that genes such as *PRELP*, *CLU*, *COMP*, *TNFRSF12A*, *INHBA*, *CILP*, and *SERPINE2*, were significantly upregulated in *CILP*+ fibroblasts. These genes are involved in promoting cell proliferation, inhibiting inflammatory pathways, and stabilizing cell structure, thereby exerting reparative and protective effects on chondrocytes. In contrast, *COL6A3*, *COL6A1*, *COL1A2*, *COL1A1*, *COL3A1*, *TGF-β1*, *MMP2*, *AEBP1*, *SPARC*, *FNDC1*, and *POSTN* were upregulated in *POSTN*+ fibroblasts. These genes may contribute to chondrocyte damage and further degeneration by promoting chondrocyte catabolism, driving inflammation, activating inflammatory pathways, and facilitating chondrocyte apoptosis and destruction.

**Conclusion:**

Our study elucidated the interactions and correlations between OA synovial cells and chondrocytes. *CILP*+ synovial fibroblasts may exert reparative and protective effects on chondrocytes of patients with OA by promoting cell proliferation, inhibiting inflammation, and stabilizing cellular structures, thereby potentially mitigating the progression of cartilage lesions in affected patients. In contrast, *POSTN*+ synovial fibroblasts may exacerbate chondrocyte deterioration in patients with OA by enhancing degradation, inflammation, and apoptosis, thereby exacerbating cartilage lesions. Investigating the underlying molecular mechanisms between OA synovial cells and chondrocytes refines the understanding of OA pathogenesis and provides valuable insights for the clinical diagnosis and treatment of OA.

## 1 Introduction

Osteoarthritis (OA) is a chronic degenerative disease with intricate pathophysiology that exerts a deleterious impact on multiple joints and joint structures throughout the body (1). Incomplete global statistics have indicated that approximately 240 million individuals worldwide suffer from symptomatic and functionally limiting OA (2, 3). The primary symptoms of OA are characterized by pain, joint stiffness, joint damage, and progressive limitation of joint mobility. These symptoms can eventually lead to disability in patients as the disease progresses. Despite the substantial expenditure on household medical care for OA treatment, the disease remains incurable (4, 5). The pathogenesis of OA is a complex process that involves the combined effects of multiple factors, including age, female sex, genetic predisposition, comorbidities, obesity, metabolic syndrome and joint injury (6, 7). The initial pathological alterations associated with OA originate in the articular cartilage. Chondrocyte proliferation, the only cell type in articular cartilage, is significantly constrained by the loss of the extracellular matrix. However, as OA progresses, pro-inflammatory factors stimulate the continuous production of matrix metalloproteinases, leading to extensive matrix degradation and resulting in the massive apoptosis of chondrocytes, which in turn causes further extracellular matrix region loss (8–10).

Furthermore, the majority of patients with OA symptoms often present with synovitis at the cellular level (11). Synovitis has been demonstrated to actively promote the production of pro-inflammatory factors and pain-related neurotransmitters (12). Moreover, synovitis can release factors related to cartilage destruction in OA, thereby exacerbating cartilage damage and advancing OA progression (13, 14). While synovitis facilitates synovial angiogenesis as a defense mechanism, this process accelerates the inflammatory process, leading to increased OA-related joint pain/hyperalgesia, restricted joint movement, and even stiffness (15). Meanwhile, fibroblasts, the main cellular component of the synovium, would undergo abnormal proliferation in response to inflammatory stimuli, promoting macrophages to produce tumor necrosis factor-α (TNF-α), ultimately resulting in a vicious cycle of inflammation (16–18).

Nevertheless, although synovitis contributes to chondrocyte damage and the progression of OA lesions, research has suggested that synovitis cannot be considered a trigger for primary OA (19). The complex network of interactions between synovial cells and chondrocytes in OA remains poorly understood and requires further elucidation (8, 20). Therefore, further investigation of the potential molecular mechanisms between OA synovial cells and chondrocytes and accurate identification of their interactions and relationships can improve our understanding of the pathogenesis of OA.

Single-cell sequencing is a powerful research method that allows for the clustering of cells to study differences in gene expression and cellular progression across different groups (21, 22). Despite the presence of various cell types, including fibroblasts, T cells, effector chondrocytes, and reparative chondrocytes, within synovial cells and chondrocytes, single-cell sequencing can provide high-resolution analysis of the correlation between these two types of cells.

This study utilized public databases for single-cell sequencing and differential expression analysis of OA to explore the relationship between OA synovial cells and chondrocytes. In addition, the study adopted cell interaction techniques to analyze the cellular interactions between the two, aiming to better understand the cellular interactions between synovial cells and chondrocytes during the onset and progression of OA. Through the investigation, our research may help elucidate the potential molecular mechanisms between OA synovial cells and chondrocytes, thereby furthering our understanding of the pathogenesis of OA.

## 2 Methods and materials

### 2.1 Acquisition and collection of single-cell RNA sequencing data

In this study, the human OA dataset GSE152805 from the GEO database was used (23). The dataset used in this study is available at www.ncbi.nlm.nih.gov/geo. The ScRNA-seq dataset of GSE152805 was generated using the 10× Genomics platform from three knee joint OA patients. These datasets were obtained using the GPL20301 platform. The dataset includes synovial tissue samples and cartilage tissue samples from three patients, with the cartilage tissue samples divided into damaged and relatively undamaged categories. A total of 10,640 synovial tissue cells and 26,192 cartilage tissue cells were collected in this dataset.

### 2.2 Integration of single-cell transcriptomes

All scRNA-seq count matrices used in this study were obtained from the GEO database. The wildly applied “Seurat” package in R (version: 4.1.0) was used for integration, analysis, and visualization of scRNA-seq data. Before integration, several processes were performed separately for each dataset, including quality control and cell selection, data normalization, and identification of highly variable features. The same package was also used to integrate the data to better understand the cellular composition and development mechanisms of OA. A cross-dataset of cells in a matched biological state was identified. Technical differences between the datasets were also corrected. Subsequently, two datasets were integrated together for subsequent analysis. Human data from different patients and tissues were also integrated to correct for sample differences using the same approach. The data were then analyzed according to the standard processing workflow in “Seurat”, including data scaling, linear (PCA), and non-linear (UMAP) dimensional reduction, as well as cell clustering.

### 2.3 Analysis of single-cell data

Single-cell raw data were obtained from published studies and analyzed using Seurat in R. The complete workflow included: setting up the Seurat Object, standard pre-processing workflow; normalizing the data, identification of highly variable features, scaling the data, performing linear dimensional reduction, determining the “dimensionality” of the dataset, clustering cells, running non-linear dimensional reduction (UMAP/tSNE), identifying differentially expressed features (cluster biomarkers), assigning cell type identity to clusters, and visualization.

### 2.4 Cell type identification

Based on reported marker genes for various cell types in the literature and cell type biomarkers mentioned in the original papers (24), we identified the following cell types and representative marker genes: Fibroblasts (*MGP*, *COL1A1*, *COL6A2*), T cells (*CD3E*, *CD3D*, *CD3G*), Dendritic cells (*FCER1A*, *CD1C*, *CD1E*), Macrophages (*C1QA*, *C1QB*, *C1QC*), B cells (*BLNK*, *CD79A*, *CD79B*), Mast cells (*TPSAB1*, *CPA3*, *RGS13*), Smooth muscle cells (*RGS5*, *ACTA2*, *TAGLN*), Endothelial cells (*TM4SF1*, *PLVAP*, *DARC*), Homeostatic chondrocytes (*MMP3*, *CHI3L1*, *CFH*), Hypertrophic chondrocytes (*CRISPLD1*, *CHRDL2*, *FRZB*), Pre-hypertrophic chondrocytes (*CLEC3A*, *C2orf82*, *FGFBP2*), Reparative chondrocytes (*COL2A1*, *SPARC*, *CTHRC1*), Pre-Fibrochondrocytes (*TNFAIP6*, *SERPINE2*, *ABI3BP*), Fibrochondrocytes (*COL1A1*, *TMSB4X*, *PRG4*), Regulatory chondrocytes (*CHI3L2*, *IFITM3*, *VCAM1*).

### 2.5 Visualization of single-cell data

Data visualization in this study was also performed using Seurat and included UMAP scatter plots, violin plots, dot plots, volcano plots, and heatmaps. UMAP scatter plots illustrate the distribution of each cell on spatial coordinates based on different gene expressions, where cells with similar expression patterns are closer in spatial distance. Violin plots display the expression levels of a specific gene or a class of genes in a particular cell type. Dot plots use color to indicate the average expression level of a gene in a particular cell type, with darker colors indicating higher expression; dot size reflects the positive expression proportion of a gene in a particular cell type, with a larger diameter indicating a higher positive proportion. Volcano plots illustrate differentially expressed genes in a specific cell type compared to others, highlighting upregulated and downregulated genes. Heatmaps compare the average expression levels of certain genes across different cell types.

### 2.6 Cell interaction analysis

The “CellphoneDB” tool, as previously reported, was used for cell interaction analysis. CellphoneDB is a publicly available repository containing information on receptors, ligands, and their interactions in human tissues. This tool, which integrates Python and R languages, enables analysis of interactions between different cell types in single-cell data. By providing CellphoneDB with the required matrix files and cell type annotation files, cell interaction analysis was conducted following the default standard procedures and parameters of CellphoneDB (25).

### 2.7 Gene enrichment analysis

Differentially expressed genes in the clusters were identified using “Seurat”. The lists of upregulated or downregulated genes in the clusters were uploaded to the online tool “ DAVID”, an online functional annotation tool that offers resources for annotation, visualization, and integrated discovery. DAVID provides researchers with a set of tools to interpret the biological significance behind large gene lists. KEGG gene enrichment analysis was subsequently performed to identify relevant gene signaling pathways based on the provided gene lists.

## 3 Results

### 3.1 Single-cell transcriptomic analysis of human OA synovial cells

**Figure 1:**
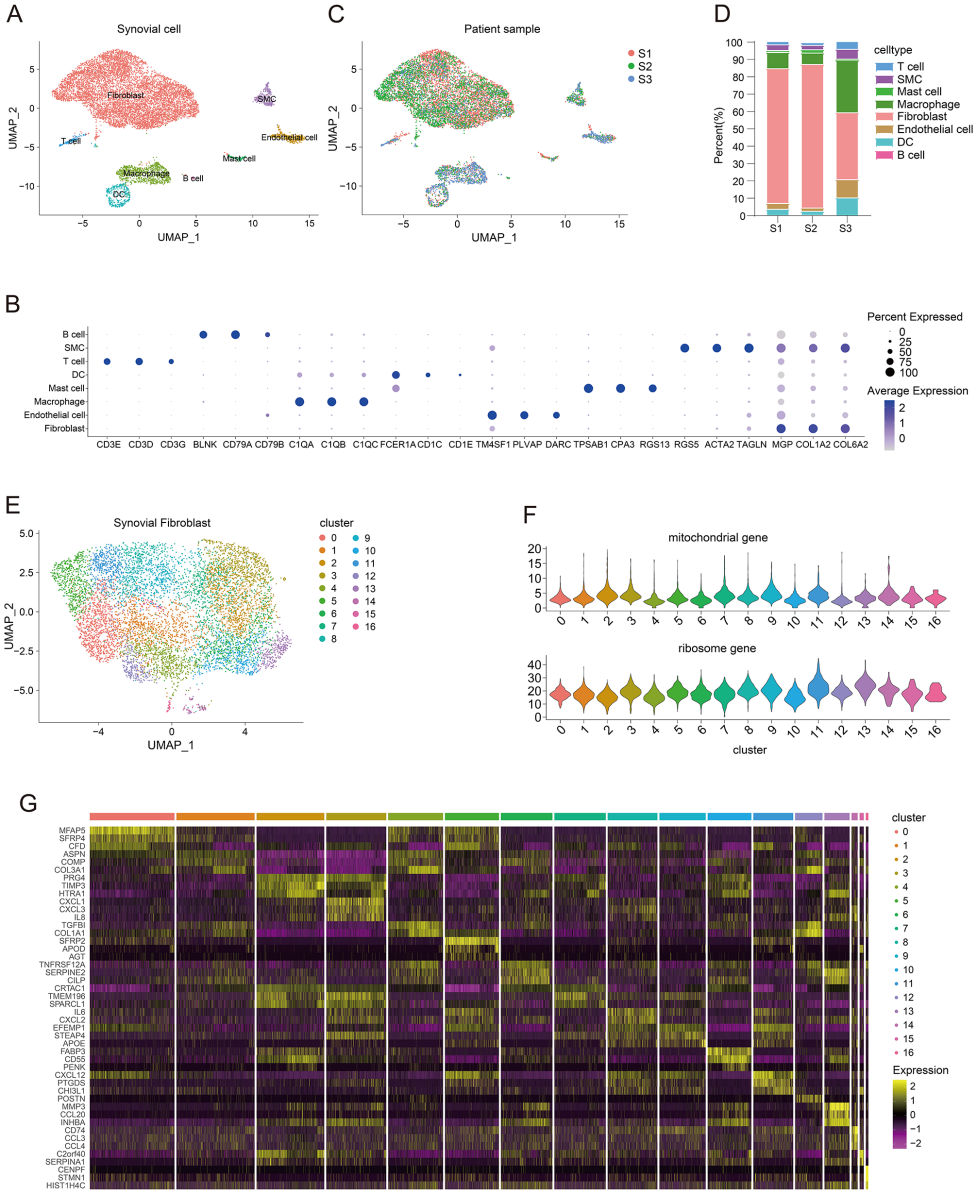
Single-cell transcriptomic analysis of human osteoarthritis synovial tissues and fibroblast clusters. **(A)** UMAP plot showing the clustering distribution of synovial tissue cells. **(B)** Dotplot displaying the marker genes for all cell types. **(C)** UMAP plot demonstrating the clustering distribution of synovial tissue cells from different patients (S1: males, S2 and S3: females). **(D)** Bar plot comparing the proportions of all types of synovial cells in different patient samples (S1: males, S2 and S3: females). **(E)** UMAP plot presenting the clustering distribution of fibroblast clusters. **(F)** Violin plot showing the proportions of mitochondrial genes and ribosomal genes in all fibroblast clusters. **(G)** Heatmap displaying marker genes for all fibroblast clusters.

To analyze single-cell sequencing data from OA synovial cells, we utilized the OA single-cell dataset GSE152805. This dataset includes human samples comprising three OA synovial cell samples and seven OA chondrocyte samples, all generated using the 10× Genomics platform. After applying the Seurat package in R for standard single-cell analysis workflow, the human OA synovial cells underwent UMAP dimensionality reduction and clustering. We identified eight different cell types: Fibroblasts (*MGP*, *COL1A2*, *COL6A2*), T cells (*CD3E*, *CD3D*, *CD3G*), Dendritic cells (*FCER1A*, *CD1C*, *CD1E*), Macrophages (*C1QA*, *C1QB*, *C1QC*), B cells (*BLNK*, *CD79A*, *CD79B*), Mast cells (*TPSAB1*, *CPA3*, *RGS13*), Smooth muscle cells (*RGS5*, *ACTA2*, *TAGLN*), and Endothelial cells (*TM4SF1*, *PLVAP*, *DARC*) (**Figure 1A**). The dot plot illustrates the marker genes for each cell type (**Figure 1B**). To evaluate the degree of integration between different samples, we compared the OA synovial cell clusters of male patients (S1) and female patients (S2, S3). The results showed that after integration, the single-cell samples of each patient were evenly distributed in the UMAP plot (**Figure 1C**). Furthermore, to compare the cellular composition differences among different patients, we presented the proportions of cell-types in different samples using bar plots. The results showed that the proportion of fibroblasts was relatively higher in both male patients (S1) and female patients (S2, S3) (**Figure 1D**). To further analyze synovial fibroblasts, we performed additional dimensionality reduction and clustering on the fibroblasts and identified 17 distinct fibroblast clusters (**Figure 1E**). The violin plot displays the proportions of mitochondrial genes and ribosome genes in all fibroblast clusters (**Figure 1F**). Moreover, we analyzed the differentially expressed genes within each fibroblast cluster, and the marker genes in the 17 clusters were presented in a heatmap (**Figure 1G**).

### 3.2 Single-cell data analysis of human osteoarthritis chondrocytes

**Figure 2:**
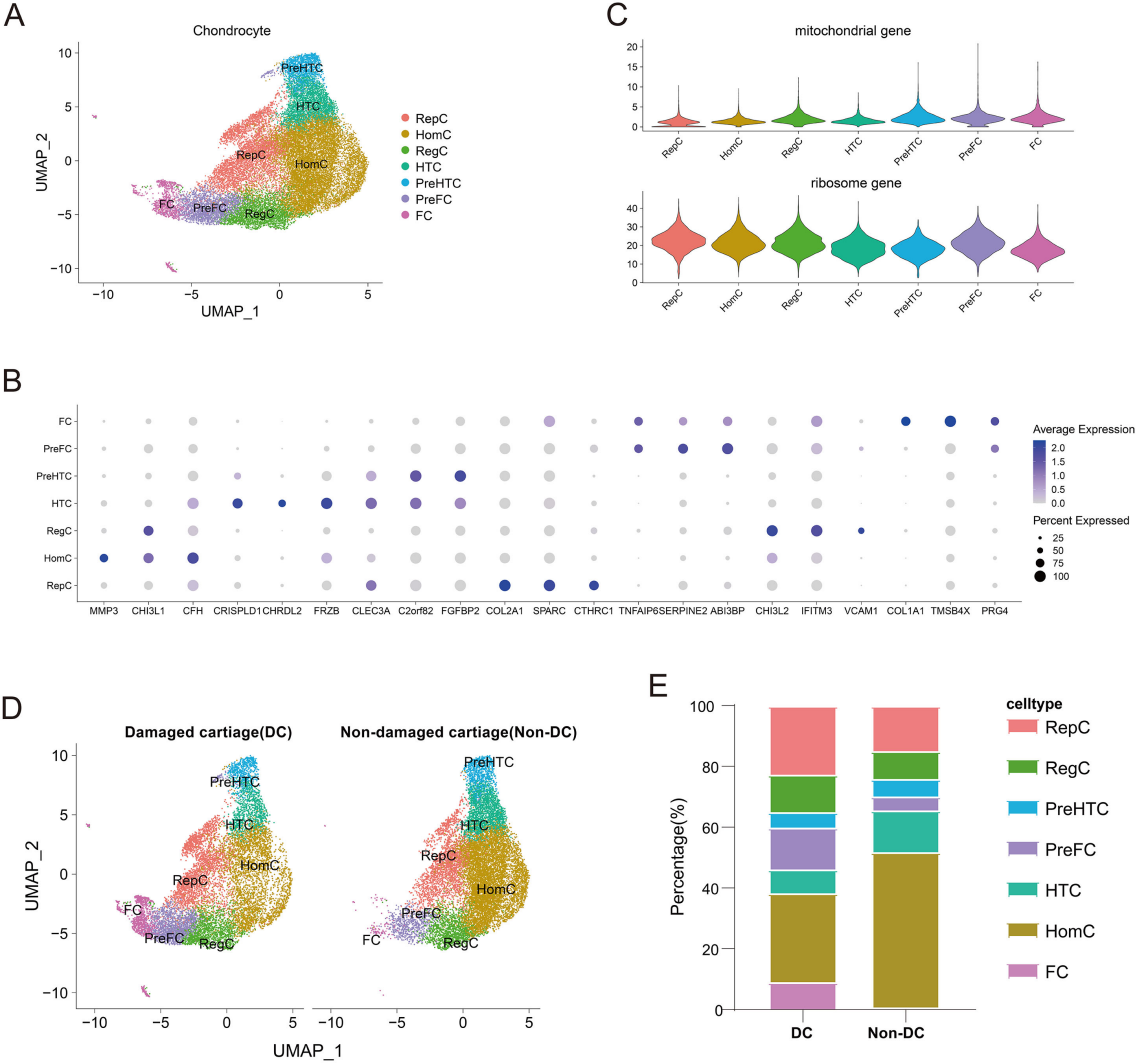
Single-cell transcriptomic analysis of human osteoarthritis chondrocytes. **(A)** UMAP plot showing the clustering distribution of chondrocytes. **(B)** Dotplot displaying the marker genes for all chondrocyte clusters. **(C)** Violin plot showing the proportions of mitochondrial genes and ribosomal genes for all chondrocyte clusters. **(D)** UMAP plot comparing the clustering distribution of cells in damaged and non-damaged cartilages. **(E)** Bar plot comparing the percentages of different clusters in damaged and non-damaged cartilages.

Pathological changes in OA are closely associated with progressive loss and destruction of joint cartilage. Therefore, studying cartilage is essential for understanding OA. For this purpose, we extracted the chondrocyte dataset from GSE152805 for single-cell sequencing analysis. Seven unique chondrocyte types were identified (**Figure 2A**), including Homeostatic chondrocytes (HomC) (*MMP3*, *CHI3L1*, *CFH*), Hypertrophic chondrocytes (HTC) (*CRISPLD1*, *CHRDL2*, *FRZB*), Pre-hypertrophic chondrocytes (preHTC) (*CLEC3A*, *C2orf82*, *FGFBP2*), Reparative chondrocytes (RepC) (*COL2A1*, *SPARC*, *CTHRC1*), Pre-Fibrochondrocytes (preFC) (*TNFAIP6*, *SERPINE2*, *ABI3BP*), Fibrochondrocytes (FC) (*COL1A1*, *TMSB4X*, *PRG4*), and Regulatory chondrocytes (RegC) (*CHI3L2*, *IFITM3*, *VCAM1*) (**Supplementary Figure S1**). Dot plots display the marker genes for each type of chondrocytes (**Figure 2B**). The violin plot shows the proportions of mitochondrial genes and ribosome genes for each chondrocyte cluster (**Figure 2C**). Next, to validate the presence of differences in chondrocyte types between injured and uninjured cartilages, we performed additional dimensionality reduction and clustering. The proportions of different chondrocyte types in both types of cartilage are displayed in a bar plot (**Figures 2D, E**).

### 3.3 Interactions between synovial fibroblast clusters and chondrocyte clusters in different types of cartilages

**Figure 3:**
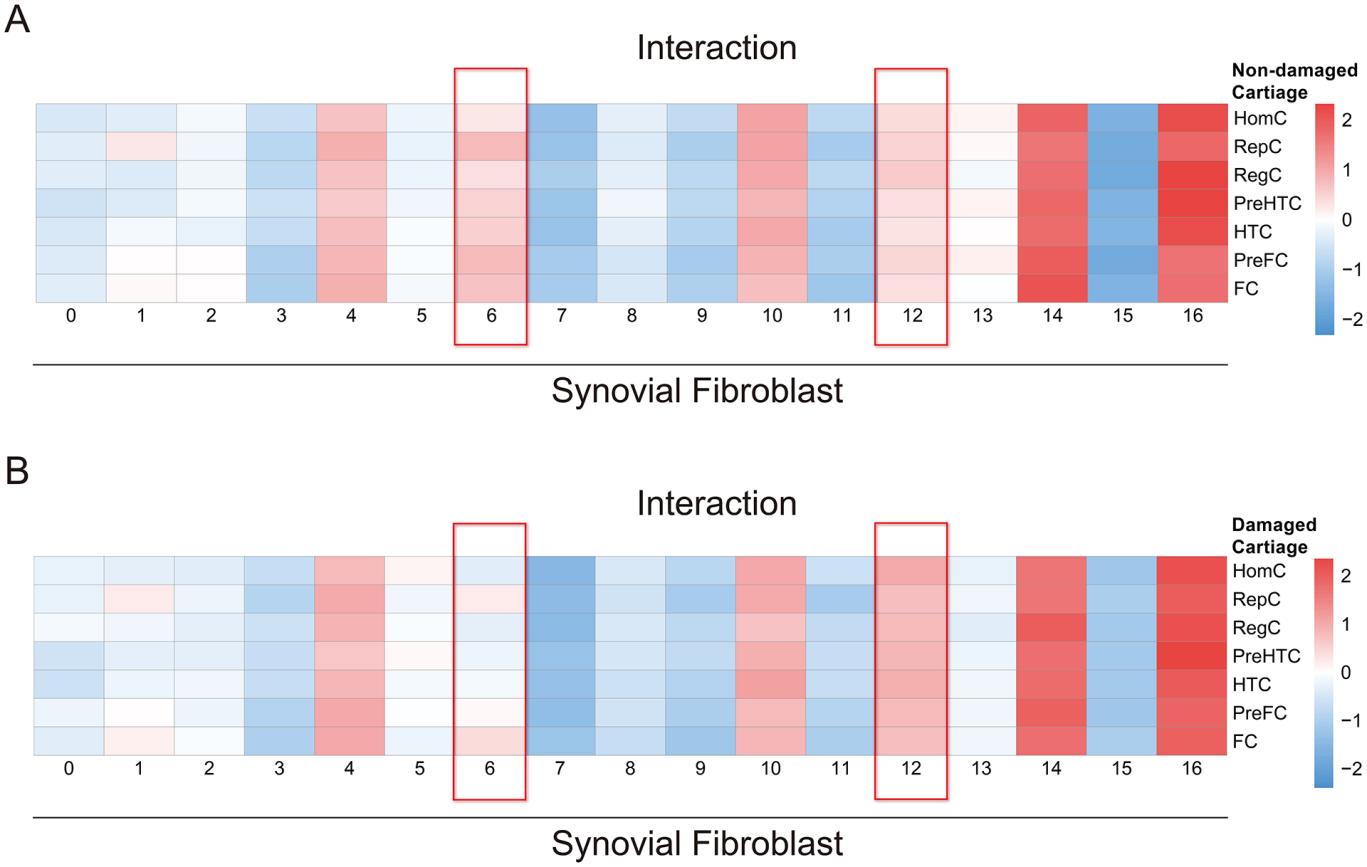
Cell-cell interactions between synovial fibroblast clusters and chondrocyte clusters. **(A)** Heatmap illustrating the intercellular interactions between synovial fibroblast clusters and cell clusters in the non-damaged cartilage. **(B)** Heatmap illustrating the intercellular interactions between clusters of synovial fibroblasts and cell clusters in the damaged cartilage.

To investigate the cellular interactions between synovial fibroblasts and different chondrocytes, we examined the cell-cell interactions among various fibroblast clusters and different types of chondrocytes in both damaged and non-damaged cartilages. Expression matrices in the damaged and undamaged groups were extracted separately. The CellphoneDB tool was employed to analyze the interaction strengths of all synovial fibroblast clusters with these two groups of chondrocytes (25). The procedure and parameters for cell interaction analysis using CellphoneDB strictly followed the standard or default modes required by CellphoneDB. The results showed significant differences in interactions between damaged/non-damaged chondrocytes and synovial fibroblasts, demonstrating heterogeneity across different synovial fibroblast clusters and chondrocytes (**Supplementary Tables 1**, **2**). To identify specific synovial fibroblast clusters related to cartilage damage or repair, quantitative statistical analysis was conducted on the interaction strengths of these two groups. The quantitative data of interaction strengths were generated and output by the CellphoneDB (**Supplementary Tables 3**, **4**). Heatmaps were used to compare the interaction strengths between different synovial fibroblast clusters and damaged or non-damaged chondrocytes. The scale values represented the relative quantitative interaction strengths. The results demonstrated significant differences in cell-cell interactions between cluster 6 and cluster 12 of synovial fibroblasts with different types of cells in two cartilage types. Cluster 6 exhibited stronger interactions with cells in the non-damaged cartilage than with cells in the damaged cartilage. Conversely, cluster 12 demonstrated stronger interactions with cells in the damaged cartilage compared to the cells in the non-damaged cartilage. Other fibroblast clusters did not display notable differences in interaction patterns. These findings suggested that cluster 6 interacted more extensively with cells in the non-damaged cartilage, potentially contributing to the repair and protection processes of articular chondrocytes, while cluster 12 was closely associated with communication among cells in the damaged cartilage, potentially relating to chondrocyte damage and ongoing deterioration (**Figures 3A, B**).

### 3.4 Differential analysis of cluster 6 and cluster 12 of fibroblasts

**Figure 4:**
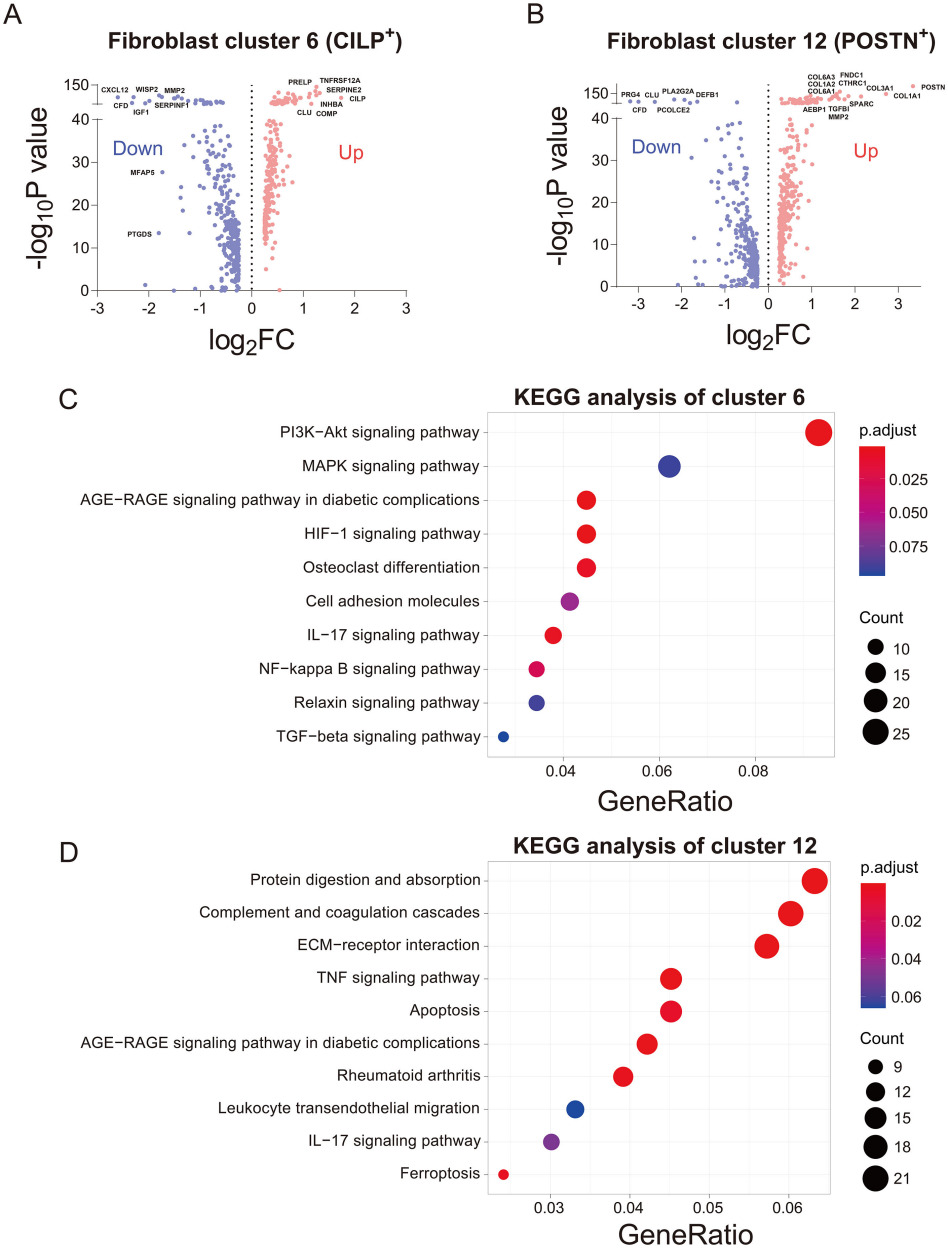
Differential gene expression analysis of damaging and reparative clusters of fibroblasts. **(A)** Volcano plot illustrating differentially expressed genes in cluster 6 of reparative fibroblasts. **(B)** Volcano plot illustrating differentially expressed genes in cluster 12 of damaging fibroblasts. **(C)** KEGG pathway enrichment analysis for fibroblast cluster 6. **(D)** KEGG pathway enrichment analysis for fibroblast cluster 12.

Through cell interaction analysis, we found that cluster 6 was associated with chondrocyte protection and repair, while cluster 12 was linked to chondrocyte damage and ongoing deterioration. In order to further validate our hypothesis, differential gene analysis was performed on fibroblast cluster 6 and cluster 12. Firstly, volcano plots were used to visualize and analyze the differentially expressed genes in both clusters. Cluster 6 exhibited high expression of genes such as *RELP*, *CLU*, *COMP*, *TNFRSF12A*, *INHBA*, *CILP*, and *SERPINE2*, with *CILP* showing the highest fold increase (**Figure 4A**). In contrast, cluster 12 showed upregulation of genes including *COL6A3*, *COL6A1*, *COL1A2*, *COL1A1*, *COL3A1*, *TGF-β1*, *MMP2*, *AEBP1*, *SPARC*, *FNDC1*, and *POSTN*, with *POSTN* being the gene with the highest fold increase in the volcano plot (**Figure 4B**). Subsequently, a comparison of signaling pathways between the two clusters was conducted, and KEGG pathway enrichment analysis revealed significant enrichment of PI3K-Akt, MAPK, and HIF-1 pathways in cluster 6 (**Figure 4C**). Conversely, cluster 12 exhibited enrichment in signaling pathways such as protein digestion and absorption, ECM receptor interaction, TNF, apoptosis, and rheumatoid arthritis (**Figure 4D**), which are commonly associated with cell apoptosis, pro-inflammation, and structural disruption.

## 4 Discussion

The pathological process of OA involves not only changes in articular chondrocytes and osteocytes but also alterations in the entire joint structure. Therefore, gaining a deeper understanding of the pathogenesis of OA and recognizing the interactions among different joint structures are crucial. In this study, through single-cell sequencing analysis, differential gene expression analysis, and intercellular interaction analysis, we identified the interactions between synovial fibroblasts and OA chondrocytes, as well as the role of fibroblasts in OA progression.

The interaction between synovial cells and chondrocytes plays a pivotal role in the pathogenesis of OA (23). Previous studies have reported the impact of synovial fibroblasts on OA chondrocytes. Fibroblasts can exhibit distinct transcriptional and epigenetic characteristics, which have unique effects on their function and the creation of different joint microenvironments (26). Genetic analysis of synovial fibroblasts in OA patients has shown upregulation of genes such as actin γ1 (*ACTG1*) and collagen genes (*COL1A1*, *COL3A1*, *COL4A1*, *COL6A3*, *COL11A1*), which promote destruction of chondrocytes and cartilage matrices through interactions between cell matrix receptors, inflammation, and degradation and metabolism, thus playing a positive role in OA progression (27, 28). In this study, we discovered that fibroblast cluster 12 exhibited high expression of multiple genes including *COL1A1*, *COL3A1*, and *COL6A3* in the cells in damaged chondrocytes. Furthermore, OA synovial fibroblasts can directly promote the activation of transforming growth factor-beta 1 (*TGF-β1*) and inhibit miR-92a expression. This leads to the activation of adenosine 5’-monophosphate (AMP)-activated protein kinase (AMPK) and p38 signaling pathways, resulting in increased expression of FOXO3 protein, inflammatory cytokines (such as TNF-α, IL-1β, vascular endothelial growth factor, and chemokine CCL2). These processes facilitate chondrocyte and cartilage injury, exacerbating the progression of OA inflammation (29–31). Similarly, in this study, we also observed elevated expression of *TGF-β1* in fibroblast cluster 12 in the cells in damaged chondrocytes.

Furthermore, normal synovial membrane function relies on fibroblasts, which are involved in the production of synovium, hyaluronic acid, collagen, and fibronectin. These substances are crucial for nourishment of chondrocytes and joint movement (32). Additionally, research has reported that normal synovial fibroblasts possess the characteristics of mesenchymal stem cells, including self-renewal and multilineage differentiation potential. They are capable of promoting bone and chondrocyte regeneration, thus exerting a protective and reparative effect on articular cartilage (33, 34). In this study, we found that fibroblast cluster 6 exhibited the most significant communication with cells in non-damaged chondrocytes, and within this cluster, multiple genes associated with pro-inflammatory processes, promotion of cartilage or chondrocyte damage and apoptosis (*MMP2*, *IGF1*, *CXCL12*, *SERPINF1*, *PTGDS*), were downregulated (35–38), consistent with previous research findings.

In our differential gene expression analysis, we found that genes such as *PRELP*, *CLU*, *COMP*, *TNFRSF12A*, *INHBA*, *CILP*, and *SERPINE2* were upregulated in cluster 6. Through literature review, we discovered that COMP stimulates chondrocyte proliferation and promotes cartilage formation and increases collagen secretion, thereby playing a reparative and protective role in chondrocytes (39–42). *CLU* has been reported to exert anti-apoptotic effects by inhibiting the activation of the p53 signaling pathway and maintaining cartilage extracellular matrix stability. It can also modulate the production of inflammatory cytokines such as IL-6 and IL-8, exhibiting anti-inflammatory properties (43–46) and thus regulating apoptosis and inflammation processes in OA. Additionally, *CILP* has been shown to play a crucial role in maintaining cartilage structure and exerting specific functions by balancing intracellular and extracellular protein homeostasis, and stabilizing chondrocyte structure in OA tissue repair (47–49). KEGG pathway enrichment analysis also showed that the high expression of cluster 6 was associated with pathways related to cell proliferation, anti-apoptosis, and promotion of the cell cycle (50–53). In contrast, cluster 12 exhibited high expression of genes such as *COL6A3*, *COL6A1*, *COL1A2*, *COL1A1*, *COL3A1*, *TGF-β1*, *MMP2*, *AEBP1*, *SPARC*, *FNDC1*, and *POSTN*. These genes have been reported to accelerate cellular aging, promote cartilage degradation, and worsen OA through mechanisms involving inflammation, and cartilage catabolism (54–56). Relevant studies have revealed that COL3A1 promotes OA-related inflammatory signaling pathways through its involvement in extracellular matrix (ECM) receptor interaction, cytokine-cytokine receptor interaction, TNF signaling pathway, and chemokine signaling pathway (57). *TGF-β1*, as a key mediator in cartilage injury and OA development, aggregates pro-inflammatory IL-18 or IL-18 receptor in OA synovial fluid, inducing chondrocyte inflammation and promoting chondrocyte catabolism (58, 59). Upregulation of matrix metalloproteinase 2 (*MMP2*) promotes cartilage degradation in OA (60). Furthermore, the high expression of core cartilage-related genes in OA (*COL1A1*, *COL3A1*, *COL1A2*, *COL5A2*, *COL2A1*, *COL6A2*, *COL7A1*) induces narrowing and disorganized collagen fibers, chondrocyte swelling, and inflammatory responses, exerting negative effects in OA pathology (61). Additionally, *POSTN* is involved in Wnt signaling activation and MMP-13 expression and contributes to cartilage degeneration through upregulation of MMPs (62). These findings suggested that *CILP*+ fibroblasts may be associated with chondrocyte repair and protection. They might exert reparative and protective effects on chondrocytes by upregulating related reparative and protective genes and signaling pathways. Conversely, *POSTN*+ fibroblasts may exacerbate chondrocyte damage and further deterioration by upregulating pro-inflammatory genes and related apoptotic and inflammatory signaling pathways. However, the specific mechanisms require further investigation and validation. In normal joints, fibroblasts promote chondrocyte proliferation or repair, serving a protective role for cartilage. In OA, however, the repair function of fibroblasts is inhibited, while pro-inflammatory, degradative, and apoptotic mechanisms are upregulated, exacerbating chondrocyte damage and accelerating OA progression.

Our study has several limitations. First, the sample size in the human OA genetic database is limited. Increasing the sample size in future studies could enhance the credibility of our conclusions. Second, although we identified interactions and relationships between OA synovial cells and chondrocytes, the specific molecular mechanisms underlying these interactions require further research and validation. Current technology does not yet allow these processes to be fully isolated and evaluated, which prevents us from performing corresponding experiments for in-depth investigation.

## 5 Conclusions

In summary, this study, employing single-cell sequencing, cell interaction, and differential gene expression analyses, revealed that certain synovial fibroblast clusters can promote joint chondrocyte damage or death through mechanisms such as pro-inflammation, catabolic metabolism, and apoptosis, thereby accelerating OA progression. As a critical component of joint structure, the synovium plays a significant role in the development of OA. This research lays a solid foundation for a better understanding of the potential molecular mechanisms between the synovium and OA progression, deepening insights into OA pathogenesis and potentially informing future clinical diagnosis and treatment strategies for OA.

## Data Availability

The original contributions presented in the study are included in the article/**Supplementary Material**. Further inquiries can be directed to the corresponding authors.
