# Supplementary figures and images for: Single-cell profiling uncovers synovial fibroblast subpopulations associated with chondrocyte injury in osteoarthritis
Source: Front Endocrinol (Lausanne). 2024 Dec 10;15:1479909. doi: 10.3389/fendo.2024.1479909 (PMC11666364; doi:10.3389/fendo.2024.1479909)

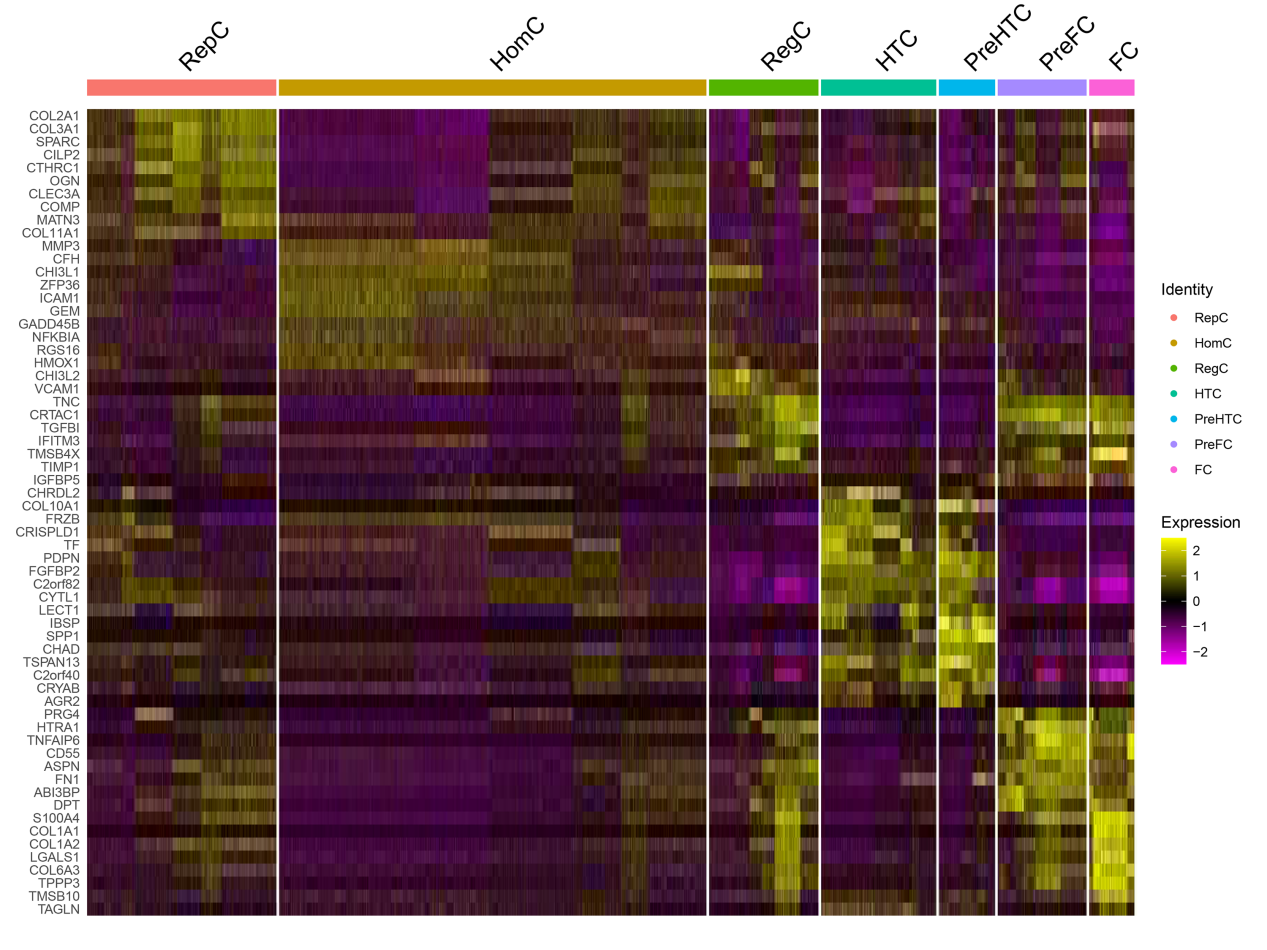


**Supplementary Figure S1.** HeatMap showing marker genes for all chondrocyte subsets.

Supplement: Supplementary file 1 [file DataSheet1.zip › Supplementary materials/Figure S1.docx]
